# Sports atmosphere and psychological resilience in college students: mediating role of growth mindset

**DOI:** 10.3389/fpsyg.2025.1532498

**Published:** 2025-04-09

**Authors:** Weijie Zhang, Jingjing Li

**Affiliations:** ^1^Physical Education College, Shanghai University, Shanghai, China; ^2^Shanghai Cao Yang No. 2 High School, Shanghai, China; ^3^Physical Education Research Center, Shanghai University, Shanghai, China

**Keywords:** sports atmosphere, psychological resilience, growth mindset, college students, mediation effect

## Abstract

**Introduction:**

The mental health of college students has garnered increasing attention currently, and psychological resilience is recognized as a crucial factor in coping with pressure and challenges. However, the influencing factors of psychological resilience require further exploration. This study aims to investigate the relationship between sports atmosphere and psychological resilience among college students, as well as the mediating role of a growth mindset.

**Methods:**

A questionnaire survey was conducted with 315 college students using the Outdoor Sports Atmosphere Scale, Growth Mindset Scale, and Brief Resilience Scale.

**Results:**

The findings revealed significant positive correlations between sports atmosphere, growth mindset, and psychological resilience. Regression analysis showed that a positive sports atmosphere significantly predicted both psychological resilience (β = 0.371, *t* = 8.648, *p* < 0.01) and growth mindset (β = 0.462, *t* = 10.227, *p* < 0.01). Structural equation modeling further confirmed that growth mindset played a significant mediating role in the relationship between sports atmosphere and psychological resilience [β = 0.182, 95% CI (0.036, 0.189), *p* < 0.05].

**Discussion:**

These results highlight the importance of a supportive sports atmosphere in fostering psychological resilience among college students, with growth mindset acting as a key intermediary factor. By cultivating a growth mindset, students may better translate the benefits of an encouraging sports atmosphere into improved psychological resilience and adaptability. These insights provide actionable strategies for educators and policymakers to design interventions that promote psychological wellbeing.

## 1 Introduction

With the acceleration of the pace of life in modern society and the increasing academic pressure, mental health issues among college students have become increasingly prominent, with widespread occurrences of depression, anxiety, and excessive stress (Xu et al., 2018). Studies have shown that students with higher levels of psychological resilience are better equipped to mitigate the above psychological issues (Lau, 2022; Nishimi et al., 2022; Poole et al., 2017) and demonstrate enhanced coping abilities under academic pressure, which contributes to improved academic performance (Shengyao et al., 2024). Psychological resilience encompasses essential components such as self-confidence, recovery resilience, optimism, and perseverance. As an individual's capacity to adapt and thrive in the face of adversity, psychological resilience often serves as a crucial protective factor against mental health issues. It often plays a critical role for college students in navigating both academic and life challenges, enhancing their self-efficacy and emotional management skills, which in turn supports their overall development (Sisto et al., 2019). However, many college students struggle with psychological distress, which can hinder their overall development. Given the increasing concerns regarding students' mental health, identifying effective strategies to foster psychological resilience has become a critical research priority.

Researchers have conducted a series of empirical studies to examine methods for enhancing psychological resilience, with common strategies include mental health education and training, social support systems construction, self-efficacy improvement, and mindfulness training. Recently, the role of exercise and its associated environmental factors has garnered increasing attention. Furthermore, a significant positive correlation has been reported between psychological resilience and physical exercise (Liu, 2020). College students engaging in moderate to high levels of physical exercise exhibit markedly better psychological resilience compared to their peers who participate in minimal physical activity (Song et al., 2014), which enable them to navigate challenges more adeptly and mitigate stress-related negative emotions (Storm and Eske, 2022), thus improve mental health (Biddle and Asare, 2011). **According to the neurobiological model of resilience**, physical exercise can also foster psychological resilience and enhance mental health by improving brain structure and function and strengthening self-regulation when facing psychological issues (Belcher et al., 2021; Mandolesi et al., 2018). These studies underscore the vital role of physical exercise in enhancing psychological resilience and improving the mental health of college students. However, some scholars argue that strengthening psychological resilience requires supportive feedback, such as social interaction and positive stimulation. This perspective suggests that mere participation in sports may have a limited impact on the improvement of psychological resilience. As a significant environmental factor, sports atmosphere may play a more pivotal role than sports participation alone in providing a comprehensive support system and psychological resources. Sports atmosphere encompasses an individual's overall perception of sports-related support, participation, social interaction, and cultural context within a specific environment (Smith et al., 2008). It includes not only the sports facilities and activity resources provided by educational institutions but also the psychological and emotional environment fostered by social support systems, such as peers, teachers, and parents. **According to Self-Determination Theory**, an individual's intrinsic motivation and satisfaction of psychological needs (autonomy, competence, and relatedness) are key factors in promoting mental health and resilience. Exercise, especially physical activity in a positive sports atmosphere, can meet these basic psychological needs, thereby enhancing an individual's self-regulation and mental resilience (Ryan and Deci, 2000). In fact, a positive sports atmosphere cultivates friendships and social networks, offering strong social and emotional support. It enhances self-efficacy, reduces negative emotions like isolation and helplessness, strengthens psychological resilience, and boosts self-confidence and overall wellbeing (Guo et al., 2024; Xu et al., 2021; Yao et al., 2022). By positively influencing individual's sports experiences, social interactions, and emotional feedback, the sports atmosphere can indirectly enhance psychological resilience and promote the development of mental health.

Psychological resilience encompasses the capacity to manage stress, regulate emotions, and adapt to change (Masten and Obradovic, 2006). Growth mindset refers to the belief that one's abilities and intelligence can be developed through effort, learning, and experience (Dweck, 2006). Key components of psychological resilience include persistence and the cognitive trait of viewing failure as a learning opportunity, both of which are integral to the growth mindset (Dweck, 2006). Research indicates that interventions promoting a growth mindset can significantly enhance the psychological resilience of adolescents in both academic and athletic contexts. According to Dweck's (2006) theory of growth mindset, individuals who believe that their abilities can be improved through effort and learning are more likely to exhibit persistence and optimism when confronted with challenges. When students embrace the notion that competence can be cultivated through hard work, they demonstrate increased resilience and stress resistance in the face of academic and athletic difficulties (Hecht et al., 2021). Thus, as a constructive cognitive framework, the growth mindset may serve as a conduit between sports atmosphere and psychological resilience. A supportive yet challenging sports atmosphere nurtures a growth mindset, boosting students' confidence to confront challenges and equipping them with the resilience needed to navigate uncertainties in both academic and life.

Currently, most existing studies concentrate on the direct relationship between sports and mental health. However, there is still insufficient discussion regarding the specific mechanisms involving sports atmosphere, growth mindset, and psychological resilience, particularly among Chinese college students. Therefore, this study aims to investigate how a positive sports atmosphere within the Chinese context can enhance psychological resilience and improve the mental health of college students by influencing their growth mindset. Through empirical research, we aspire to provide policy recommendations and practical strategies for schools, families, and society to promote the psychological resilience of college students, ultimately achieving a comprehensive improvement in their mental wellbeing.

## 2 Literature review

### 2.1 Psychological resilience

Psychological resilience is an internal trait that encompasses the interactions among cognition, emotion, and behavior. It is frequently regarded as a positive psychological resource that enables individuals to cope with adversity while fostering personal growth and development (Gloria and Steinhardt, 2016; Yin et al., 2023). Individuals with high psychological resilience tend to recover more swiftly from changing situations and may even experience “post-traumatic growth.”

Although research on college students' psychological resilience has yielded many valuable finding, such as the significant enhancement of self-efficacy, reduction of academic burnout, and improvement of emotional regulation (Cai et al., 2023; Gong et al., 2023; Guo et al., 2022), several areas remain ripe for further exploration. Previous studies have primarily regarded psychological resilience as an originating variable to investigate its direct or indirect mechanisms on specific psychological traits or behavioral outcomes, including its relationship with academic performance improvement, psychological wellbeing, and social skills (Dong et al., 2024; Li and Guo, 2023; Tanji et al., 2021). However, research on the origins and core effects of psychological resilience remains limited, leading to an incomplete understanding of the mechanisms that effectively enhance resilience among college students.

#### 2.1.1 Environmental factors and psychological resilience

**Environmental Interaction Theory** emphasizes the dynamic interaction between individuals and their environments, highlighting the environment as a crucial factor influencing individual behavior. Individuals can continuously adapt their behavior through their interactions with the environment (Ntoumanis et al., 2021). **Self-Determination Theory** asserts that autonomy, competence, and relatedness are fundamental psychological needs present in any social context (Flannery, 2017). These factors play a crucial role in shaping and strengthening psychological resilience. When these needs are met, the social environment nurtures intrinsic motivation and facilitates the internalization of extrinsic motivation. This process enhances the sense of fulfillment and wellbeing derived from participation in various activities, ultimately fostering positive and healthy personal development (Yin et al., 2023). Both theories highlight the profound influence of the environment on human behavior.

An increasing number of studies indicate that environmental factors could significantly influence the psychological resilience among college students. Multi-level environmental systems involved in individual development-including microsystems (such as family and school), mesosystems (such as community organizations), and macrosystems (such as culture and social policies), and each play a role in shaping an individual's psychological resilience (Luo et al., 2023; Ungar and Theron, 2020). The family, campus, and social environments are critical factors influencing the psychological resilience of college students. Within the family context, strong emotional support contributes to stability under pressure, while positive parent-child interactions enhance resilience (Ao et al., 2025). Furthermore, a higher economic status alleviates financial stress, thereby improving coping abilities (Goh et al., 2015). On campus, access to learning resources, guidance from tutors, and support from peers help students maintain a positive attitude when facing academic challenges (Wilks and Croom, 2008). Additionally, psychological counseling and emotional support services play a vital role in emotional regulation and stress management (Hartley et al., 2022). In the social environment, community organizations, volunteer activities, and participation in clubs strengthen social support networks and foster a sense of belonging (Stewart and Yuen, 2011). Positive interpersonal relationships provide emotional and practical support, reducing loneliness (Zhou et al., 2022). Additionally, cultural atmosphere, including recognition and encouragement of a specific culture's coping abilities and positive coping strategies, can also influence psychological resilience (Weishaar et al., 2025). In conclusion, a supportive environment—providing emotional support, resources, and social acceptance—enhances belonging, security, and psychological resilience. Interaction with the environment helps individuals develop coping skills, adapt to challenges, and refine emotional regulation. Additionally, supportive feedback that reinforces progress enhances tolerance for challenges, further strengthening overall psychological resilience (Luthar and Cicchetti, 2000; Trigueros et al., 2019).

It is noteworthy that the campus serves as the most crucial environment for college students, functioning both as a space for study and for living. Recent research has focused on the impact of the campus environment on the psychological resilience of college students. Scholars increasingly acknowledge that the sports atmosphere, as a significant aspect of the campus environment, plays a vital role in enhancing psychological resilience.

#### 2.1.2 Sports atmosphere and psychological resilience

Sports atmosphere typically refers to the sports environment shaped by the surrounding community and its associated resources, which includes sports information, peer interpersonal support, and a conducive sports environment, key components of various support systems (Chen and Luo, 2016). Sports atmosphere is often regarded as an environmental factor that encompasses emotional support, encouragement, competitive spirit, team cohesion, and expectations regarding individual and collective behavior. It primarily addresses the social and cultural elements perceived by a team or individual within a sports context, as well as the influence these factors exert on participation and performance in sports. Research indicates that a positive sports atmosphere exerts a lasting and stable influence on athletic activities aimed at enhancing physical fitness, promoting health, and supporting overall development, while also encouraging healthy behaviors among adolescents (Dong et al., 2023; Liu et al., 2011; Slater et al., 2010).

A positive sports atmosphere plays a crucial role in shaping individuals' motivation, behavior, and psychological wellbeing. Self-Determination Theory (Ryan and Deci, 2000) suggests that providing athletes with opportunities for autonomous choice fosters intrinsic motivation by fulfilling their psychological need for autonomy. This, in turn, contributes to the enhancement of psychological resilience (Hodge and Gucciardi, 2015; Trigueros et al., 2019). Empirical research indicates that a supportive sports atmosphere can boost self-efficacy, encouraging individuals to adopt positive coping strategies when facing challenges, thereby improving their adaptability and resilience (Masten and Obradovic, 2006). Moreover, a favorable sports atmosphere facilitates habit formation and lifelong physical activity engagement (Zuo et al., 2023), which are associated with enhanced emotional regulation and wellbeing (Dong et al., 2022).

Beyond its impact on sports participation, a positive sports atmosphere contributes to emotional and psychological wellbeing by reducing dependence on mobile devices (Li and Dong, 2022), alleviating negative emotions (Wang, 2019), and fostering peer relationships that enhance subjective well-being (Cheng and Jiao, 2024). Additionally, it has been shown to mitigate academic stress through the mediating effect of psychological resilience, emphasizing the vital role of physical education in developing positive psychological traits and improving academic performance (Liu et al., 2024).

Despite existing research on sports atmosphere as a mediating variable influencing exercise adherence (Liu et al., 2011), sports lifestyle, and physical activity behavior (Dong et al., 2022; Wu et al., 2023), its direct predictive role in psychological resilience requires further exploration. Given the theoretical and empirical evidence, **we propose Hypothesis 1 (H1): sports atmosphere positively predicts psychological resilience**.

### 2.2 Growth mindset, sports atmosphere and psychological resilience

Mindset theory posits the existence of two distinct types of mindsets: the fixed mindset and the growth mindset. The latter asserts that abilities and intelligence are malleable, capable of continual development through effort and learning (Yin et al., 2023). Neurobiological research supports this idea, showing that the brain possesses plasticity-its structure and function continuously adapt throughout life in response to learning and experiences. This neuroplasticity serves as the biological foundation for the development and reinforcement of a growth mindset (Zatorre et al., 2012). Moreover, a growth mindset fosters academic and professional development as well as improved social behavior (Walker and Jiang, 2022). Individuals who adopt a growth mindset are more likely to perceive difficulties and setbacks as opportunities for self-improvement, which enhances intrinsic motivation and subsequently leading to improved learning performance and personal growth (Zatorre et al., 2012). Additionally, studies have shown that a growth mindset can mitigate negative emotions stemming from frustration and may indirectly enhance psychological resilience by promoting effective coping mechanisms (Zeng et al., 2016).

Numerous studies have identified key factors influencing the development of a growth mindset in college students, emphasizing the role of growth mindset curricula, psychological education and counseling, and positive feedback as important strategies for fostering this mindset (Wolcott et al., 2021). Short-term growth mindset training programs, along with daily integration of growth mindset principles in education, have been shown to enhance learning motivation, boosts students' sense of achievement, and facilitate mindset shifts (Hecht et al., 2021; Zhang et al., 2022). Cognitive-behavioral therapy, psychological education, and academic tutoring further support students' ability to understand failure and challenges, reinforcing a growth mindset (Chen et al., 2021; Schleider and Weisz, 2016). Additionally, studies highlight that social support network, self-regulation abilities, self-efficacy, and coping strategies play a crucial role in fostering positive thinking and psychological resilience, further contributing to the internalization of a growth mindset (Masten, 2014).

Recent research suggests that a sports-oriented atmosphere may influence the formation of a growth mindset. For example, a leisure physical exercise environment has been found to predict academic stress in high school students through the mediating role of growth mindset and self-control (He et al., 2023). Among college students, factors such as self-perception in sports, teachers' feedback and encouragement, and peer performance in athletics can enhance intrinsic motivation, reinforcing the belief that abilities can improve through effort (Zhang et al., 2022). This internalization process further strengthens the development of a growth mindset (Schneider and Kwan, 2013). Thus, **we propose the Hypothesis 2 (H2): Sports atmosphere positively predicts growth mindset in college students**.

Moreover, studies indicate that when the sports atmosphere provides sufficient challenges and support, students are more likely to cultivate a growth mindset, leading to greater psychological resilience (Yun et al., 2013). Similarly, growth-oriented feedback from coaches in sports training encourages students to adopt growth-oriented thinking, ultimately enhancing their ability to cope with stress and build psychological resilience (Sarrasin et al., 2018). From the perspective of Self-Determination Theory, psychological resilience is primarily an internal psychological trait, while a positive sports atmosphere provides essential external support and challenges (Ryan and Deci, 2000). As a mediating variable, growth-oriented thinking can internalize the positive influences of the external environment into an individual's cognitive and emotional regulation capabilities, thereby effectively enhancing psychological resilience. For instance, a positive sports atmosphere fulfills students' psychological needs—such as autonomy, competence, and relatedness—by offering choices in sports activities, encouraging challenges, and fostering team interactions, which increases the likelihood of forming a growth mindset and enhances their psychological resilience in the face of pressure. Thus, a positive sports atmosphere not only increases individual engagement in physical activities but also enhances self-efficacy through successful sporting experiences and the development of athletic abilities (Li et al., 2024). This enhancement, in turn, fosters the formation and progression of a growth mindset (Cherewick et al., 2023; Zhao et al., 2023), thereby, reinforcing psychological resilience (Wiedenman et al., 2024). **Given the empirical and theoretical evidence, we propose the Hypothesis 3 (H3):** Growth mindset mediates the relationship between sports atmosphere and psychological resilience.

**Figure 1:**
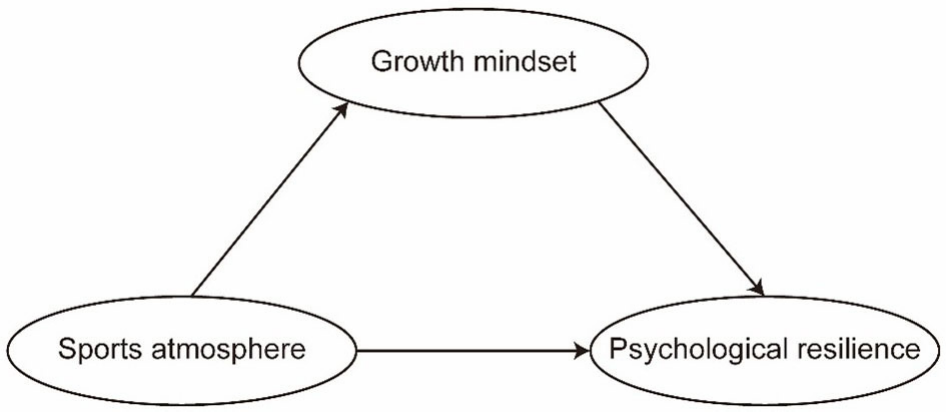
Theoretical model in this study.

Based on the literature review presented, this study proposes a hypothetical model in which sports atmosphere and growth mindset are identified as predictors of psychological resilience, with growth mindset serving as a mediator. The proposed model is illustrated in Figure 1.

## 3 Research subjects and methods

### 3.1 Research subjects and research procedure

The participants (college students currently enrolled in full-time undergraduate programs who provided informed consent) in this study were recruited from five universities in Shanghai, selected using a convenience sampling method. This method is acceptable in exploratory studies and preliminary validation models (Etikan et al., 2015) due to constraints in our resources. In addition, in the relatively new field of studying the relationship between sports atmosphere, growth mindset, and psychological resilience, convenience sampling provides theoretical and data support for subsequent larger and more rigorous random sampling studies. The researcher served as the primary examiner, distributing the electronic questionnaire during teaching classes. Questionnaires were distributed after a specific course as part of classroom activities. The researchers provided standardized guidance before issuing the questionnaire, and emphasized the anonymity and voluntary nature of the questionnaire to minimize the influence of classroom situations on the responses. We maintain a quiet environment during the questionnaire filling process and allow students to choose to fill in or opt out without any evaluation. A total of 340 questionnaires were distributed during the research, and all of which were successfully collected. The pre-test indicated that each questionnaire required approximately 8 min for completion. All participants signed an informed consent form, which outlined the study's purpose and ensured the confidentiality of the data. Furthermore, participants were explicitly informed that their responses would remain completely anonymous, that all data would solely be utilized for academic research, and that the data would be securely stored in an encrypted database accessible only to members of the research team. This study received approval from the Ethics Committee of Shanghai University (No. ECSHU 2024-101). Participants who completed the questionnaire were offered a small gift as a token of appreciation for their time and contribution, which was unrelated to the results of the questionnaires. Participants with incomplete questionnaires or inconsistent response patterns were excluded from the final analysis. After excluding invalid questionnaires due to mid-survey dropouts, and responses completed in an unreasonably short time, 315 valid questionnaires were obtained, resulting in an effectiveness rate of 92.64%. The sample comprised 171 males (54.3%) and 144 females (45.7%). The fields of study represented included science and engineering with 154 participants (48.9%), humanities and social sciences with 90 participants (28.6%), and physical education and arts with 71 participants (22.5%). Specific details of the sample selection are presented in Table 1.

**Table 1:** Distribution of demographic variables among survey participants.

**Variable**	**Category**	**Number of people**	**Percentage (%)**
Gender	Male	171	54.3
	Female	144	45.7
Major	Science and Engineering	154	48.9
	Humanities and Social Sciences	90	28.6
	Physical Education and Arts	71	22.5
Total		315	100.0

### 3.2 Research tools

#### 3.2.1 Outdoor sports atmosphere scale

The Outdoor Sports Participation Atmosphere Scale was developed by Chinese scholar Liu Weina to measure the sports atmosphere among adolescent groups (Liu et al., 2011). It primarily assesses five dimensions: interpersonal connection (e.g., “During outdoor sports, I have received help from peers”), nature connection (e.g., “Outdoor environments energize me”), interpersonal barriers (e.g., “My parents discourage me from participating in outdoor sports”), information accessibility (e.g., “When encountering difficulties in outdoor sports, I receive encouragement from others”), and conditional barriers (e.g., “My heavy academic workload leaves me no time for outdoor sports”). Responses of participants were evaluated using a 5-point Likert scale, where 1 represented “strongly disagree” and 5 represented “strongly agree.” The midpoint, “neither agree nor disagree,” was assigned a rating of 3. Higher scores on this scale indicate a more positive perception of the sports atmosphere. In this investigation, the Cronbach's α coefficient for the scale was 0.919, and the confirmatory model fit indices demonstrated favorable results (χ^2^/df = 1.239, GFI = 0.998, TLI = 0.997, IFI = 0.998, RMSEA = 0.028, RMR = 0.015).

#### 3.2.2 Growth mindset scale

The Growth Mindset Scale was developed by Dweck to examine the contrast between fixed and growth mindsets in the context of university students' academic experiences (Dweck, 2006). The scale consists of 6 items rated on a 5-point Likert scale, including 3 positively-scored items (e.g., “You can always significantly change your intelligence level”) and 3 reverse-scored items (e.g., “Intelligence is something that is difficult to change”). Responses of participants were evaluated using a 5-point Likert scale, where 1 represented “strongly disagree” and 5 represented “strongly agree.” The neutral option, “neither agree nor disagree,” was assigned a rating of 3. Higher total scores indicate a stronger tendency toward a growth mindset. In this study, the Cronbach's α coefficient for the Growth Mindset Scale was found to be 0.878. Results from confirmatory factor analysis indicated that the single-factor model exhibited good fit indices (χ^2^/df = 1.643, GFI = 0.996, TLI = 0.989, IFI = 0.996, RMSEA = 0.045, RMR = 0.016), thereby demonstrating strong reliability and validity.

#### 3.2.3 Brief resilience scale

The Brief Resilience Scale, developed by Smith and colleagues in 2008 (Smith et al., 2008), comprises six items. As validated by Chinese scholars, including Chen Wei, this scale is specifically designed to assess an individual's capacity to recover from stress, particularly health-related stress or pressure events (Chen et al., 2020). It includes three positively worded items and three negatively worded items (e.g., “I can quickly recover from setbacks,” and “I often feel overwhelmed when facing stressful events”). The scale employs a 5-point Likert scoring method, ranging from “very much not true” to “very much true,” with scores assigned from 1 to 5 points. The neutral option, “neither agree nor disagree,” was assigned a rating of 3. Higher total scores indicate a stronger psychological resilience. In this study, the Cronbach's α coefficient for the Brief Resilience Scale was found to be 0.745. Furthermore, confirmatory model fit indices demonstrated favorable results (χ^2^/df = 1.746, GFI = 0.989, TLI = 0.977, IFI = 0.991, RMSEA = 0.049, RMR = 0.024), indicating strong reliability and validity.

### 3.3 Data processing

Various statistical analyses were employed to examine our research hypotheses. The missing value in the variable was replaced with the mean of the variable. Initially, we conducted a confirmatory factor analysis (CFA) using SPSS 26.0 to assess the structural validity of the scales utilized in this study. The reliability of the scales was analyzed using Cronbach's alpha coefficient, where values exceeding 0.7 were deemed acceptable, and those above 0.8 indicated good reliability. Subsequently, we performed validity analysis, correlation analysis, and multiple linear regression analysis. Finally, we employed Amos 24.0 to construct a structural equation model that analyzed the relationships among sports atmosphere, growth mindset, and psychological resilience, sequentially verifying both direct and indirect (mediating) effects.

## 4 Results and analysis

### 4.1 Common method bias test

This study utilized self-report methods for data collection and applied Harman's single-factor test to assess common method bias. An exploratory factor analysis was conducted on all items pertaining to sports atmosphere, growth mindset, and psychological resilience. The results revealed the presence of four factors with eigenvalues exceeding 1, with the first factor explaining 38.6% of the variance, which falls short of the 40% threshold. This finding suggests that the data from this study does not exhibit a significant common method bias issue.

### 4.2 Validity analysis

To test the validity of the scales, we constructed a measurement model encompassing three latent variables: sports atmosphere, growth mindset, and psychological resilience. This model includes a total of 19 observed variables. The results of the confirmatory factor analysis (CFA) indicated that the absolute fit indices, incremental fit indices, and parsimony fit indices of the measurement model all satisfied the fit criteria, demonstrating a good fit (Table 2).

**Table 2:** Model fit indices.

**Index category**	**Index name**	**Fit criterion**	**Statistical result**	**Acceptability**
Absolute fit indices	GFI	>0.8	0.860	Acceptable
	AGFI	>0.8	0.964	Acceptable
	RMSEA	< 0.08	0.052	Acceptable
Incremental fit indices	NFI	>0.8	0.919	Acceptable
	IFI	>0.8	0.922	Acceptable
	CFI	>0.8	0.897	Acceptable
	RFI	>0.8	0.814	Acceptable
Parsimonious fit indices	CMIN/df	< 3	1.918	Acceptable
	PGFI	>0.5	0.784	Acceptable

Subsequently, we assessed the construct validity and discriminant validity of the scales. The results indicated that all three latent factors achieved satisfactory convergent validity, as evidenced by their average variance extracted (AVE) values exceeding 0.5 and composite reliabilities (CR) surpassing the 0.7 threshold (Table 3). The discriminant validity analysis demonstrated that the square roots of the AVE values (0.793 for exercise climate, 0.716 for growth mindset, and 0.679 for psychological resilience) were all greater than the maximum absolute inter-factor correlation coefficients, thereby confirming strong discriminant validity among the constructs (Table 4).

**Table 3:** AVE and CR values for constructs.

**Construct**	**Average variance extracted (AVE)**	**Composite reliability (CR)**
Sports atmosphere	0.629	0.920
Growth mindset	0.593	0.860
Psychological resilience	0.766	0.752

**Table 4:** Pearson correlations and square roots of AVE values.

**Variable**	**Sports atmosphere**	**Growth mindset**	**Psychological resilience**
Sports atmosphere	0.793^#^		
Growth mindset	0.497	0.716^#^	
Psychological resilience	0.473	0.375	0.679^#^

^#^represent the square root of the average variance extracted (AVE) values for each construct.

### 4.3 Descriptive statistics and correlation analysis among variables

Pearson correlation analysis was conducted to investigate the relationships among sports atmosphere, growth mindset, and psychological resilience. The results demonstrated a significant positive correlation among these variables (Table 5).

**Table 5:** Correlation analysis among variables.

**Variable**	**M ± SD**	**Sports atmosphere**	**Growth mindset**	**Psychological resilience**
Sports atmosphere	4.24 ± 0.78	1		
Growth mindset	4.04 ± 0.70	0.497^**^	1	
Psychological resilience	3.34 ± 0.66	0.473^**^	0.375^**^	1

^**^p < 0.01.

### 4.4 Mediating effect test

This study employed multiple linear regression analysis to investigate the influence of sports atmosphere and growth mindset on psychological resilience. All variables were standardized, and two demographic variables (gender and major) were controlled. The sports atmosphere was utilized as the predictor variable for psychological resilience and growth mindset, while the growth mindset served as the predictor variable for psychological resilience, thereby constructing three sets of regression equations. By constructing three regression models (Table 6), we examined the predictive effect of sports atmosphere on psychological resilience (Model 1), the predictive effect of sports atmosphere on growth mindset (Model 2), and the combined predictive effect of sports atmosphere and growth mindset on psychological resilience (Model 3). The results of Model 1 indicated that sports atmosphere positively predicts psychological resilience (β = 0.371, *t* = 8.648, *p* < 0.01), suggesting that a more positive sports atmosphere is associated with stronger psychological resilience. The explanatory power of this model (*R*^2^ = 0.241) indicates that the sports atmosphere accounts for ~24.1% of the variance in psychological resilience, with a significant F-test result (*F* = 32.997, *p* < 0.01), thereby validating the model. According to effect size guidelines in social science research, an R^2^ value ranging from 0.04 to 0.25 is classified as a medium effect, while values exceeding 0.25 are considered indicative of a large effect. The explanatory power of this model approaches the threshold for a large effect, suggesting that sports atmosphere is a significant predictor of psychological resilience. Results from Model 2 revealed that sports atmosphere positively predicts growth mindset (β = 0.462, *t* = 10.227, *p* < 0.01), indicating that a more positive sports atmosphere correlates with a stronger growth mindset. The model accounted for 25.4% of the variance in growth mindset, yielding a highly significant F-test result (*F* = 35.360, *p* < 0.01), thereby confirming the model's reliability. This effect size exceeds the medium effect threshold, demonstrating a relatively high explanatory power that further substantiates the potential value of the sports atmosphere in fostering growth mindset. Results from Model 3 indicated that both sports atmosphere (β = 0.283, *t* = 5.808, *p* < 0.01) and growth mindset (β = 0.192, *t* = 3.623, *p* < 0.01) serve as significant predictors of psychological resilience. This model explained 27.2% of the variance in psychological resilience, accompanied by a highly significant F-test result (*F* = 28.944, *p* < 0.01), suggesting that sports atmosphere and growth mindset collaboratively enhance psychological resilience. The joint model accounted for an additional 3.1% of the variance, surpassing the 2% threshold deemed necessary for practical significance in social science research.

**Table 6:** Regression analysis.

**Variable**	**Psychological resilience**		**Growth mindset**		**Psychological resilience**	
	β	**t**	β	**t**	β	**t**
Constant	1.933^**^	8.782	1.959^**^	8.460	1.557^**^	6.503
Gender	−0.172^*^	−2.579	0.123	1.748	−0.196^**^	−2.894
Major	0.051	1.228	−0.031	−0.722	0.057	1.399
Sports atmosphere	0.371^**^	8.648	0.462^**^	10.227	0.283^**^	5.808
Growth mindset					0.192^**^	3.623
	R^2^ = 0.241, F = 32.997^**^		R^2^ = 0.254, F = 35.360^**^		R^2^ = 0.272, F = 28.944^**^	

^*^p < 0.05, ^**^p < 0.01.

**Figure 2:**
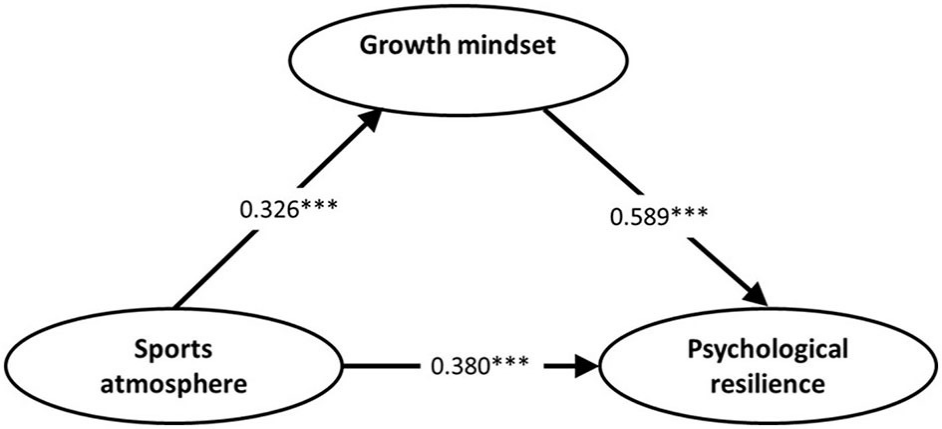
Path coefficient diagram of the mediating model. ***p < 0.001.

This finding suggests that growth mindset plays a partial mediating role between sports atmosphere and psychological resilience. In summary, the results provide robust support for the proposed model, with sports atmosphere positively influencing psychological resilience, both directly and through the mediation of growth mindset (Figure 2).

Based on the analysis conducted, a structural equation model was constructed to investigate the mediating effect of growth mindset on the relationship between sports atmosphere and psychological resilience. The analysis of the structural equation model (Table 7) yielded several key findings. Firstly, the total effect path coefficient of sports atmosphere on psychological resilience was β = 0.544, with a 95% confidence interval (CI) that excluded 0 (95% CI: 0.287–0.563; *p* < 0.01). This finding indicates that sports atmosphere significantly and positively predicts psychological resilience, thereby confirming Hypothesis 1 (H1). Secondly, the direct effect path coefficient remained statistically significant at β = 0.362 (95% CI: 0.187–0.378; *p* < 0.01), suggesting that sports atmosphere maintains its direct positive impact on psychological resilience even after considering the mediating role of growth mindset. This result supports Hypothesis 2 (H2). Thirdly, the indirect effect through growth mindset was 0.182 (95% CI: 0.036–0.189; *p* < 0.05), demonstrating that growth mindset partially mediates the relationship between sports atmosphere and psychological resilience. The significant indirect effect provides empirical support for Hypothesis 3 (H3).

**Table 7:** Test of mediating effect pathways.

**Model effect**	**β (standardized path coefficient)**	**95% CI**		**SE (Standard error)**	**p**
		**Lower**	**Upper**		
Total Effect	0.544	0.287	0.563	0.043	0.000
Direct Effect	0.362	0.187	0.378	0.049	0.000
Indirect Effect	0.182	0.036	0.189	0.039	0.023
Sports Atmosphere = >Growth Mindset	0.326	0.314	0.551	0.045	0.000
Growth Mindset = >Psychological Resilience	0.589	0.382	0.591	0.053	0.000

## 5 Discussion

Our study provides valuable insights into the effects of sports atmosphere on psychological resilience and the role of growth mindset. Using a structural equation model, we find that sports atmosphere significantly predicts psychological resilience of college students, with a growth mindset serving as a crucial mediator. These findings offer valuable directions and strategies for enhancing psychological resilience among college students.

### 5.1 Effects of sports atmosphere on psychological resilience

The findings in the present study indicate that sports atmosphere positively predicts psychological resilience (β = 0.371, *t* = 8.648, *p* < 0.01), consistent with previous research showing that both a positive sports atmosphere and physical exercise behaviors contribute to students' psychological resilience (Dong et al., 2023; Zhao et al., 2024). According to Self-Determination Theory, a positive sports atmosphere, including an individual's holistic perception of support, engagement, social interaction, and cultural ambiance in sports-related contexts (Smith et al., 2008), can significantly contribute to the satisfaction of individual's psychological needs, namely autonomy, competence, and relatedness, serve as intrinsic motivators for their actions (Ryan and Deci, 2000), thereby enhancing psychological resilience. For example, when coaches provide athletes with sufficient autonomy during training, athletes are more likely to experience intrinsic motivation and strengthen their psychological resilience (Hodge and Gucciardi, 2015; Trigueros et al., 2019). Research indicates that a dynamic and inclusive team culture, robust support among teammates, and the consideration of members' psychological needs during goal-setting collectively foster identification with team values, enhance team cohesion, promote autonomy, and ultimately strengthen athletes' psychological resilience (Gu et al., 2022). Additionally, a positive sports atmosphere at universities can enhance the flow experience of college students during physical activities (Kong, 2017) and significantly bolsters their psychological resilience (Mao et al., 2024). Collectively, these studies align with our findings, reinforcing the notion that a positive sports atmosphere is instrumental in enhancing psychological resilience among college students.

### 5.2 Role of growth mindset in sports atmosphere and psychological resilience

According to mindset theory, mindsets are classified into two categories: fixed mindsets and growth mindsets, the latter can be shaped by multiple factors, including social, cultural, economic, and environmental elements (Yang et al., 2023). Among them, a supportive campus environment, such as a positive classroom culture, effective school management, and strong institutional support, play a pivotal role of in cultivating a growth-oriented mindset (Yu, 2017). Additionally, educational approaches, such as interdisciplinary curriculum design, interactive online-offline learning, and a student-centered ecological approach contribute to the development of growth mindset. Even simple interventions, like positive-language posters, can influence the mode of mindset among students (Li and Geng, 2018). All the above researches emphasize the important role of environmental factors in shaping growth mindset. Campus sports atmosphere, as an important component of campus environment, should also be an important factor in cultivating students' growth mindset. However, these views have not been reported in previous studies. In our present study, the results indicate that a positive sports atmosphere significantly predicts the development of growth mindset among college students (β = 0.462, *t* = 10.227, *p* < 0.01). This can be interpreted from multiple psychosocial perspectives. According to Self-Determination Theory, autonomy-supportive sports environments, which provide students with the freedom to choose physical activities, design their own training plans, and participate in decision-making during team sports, have been shown to enhance feelings of autonomy and respect. These experiences, in turn, foster intrinsic motivation and long-term adherence to exercise, enabling students to embrace challenges and maintain a constructive attitude when encountering setbacks (Teixeira et al., 2012). The sense of control and self-direction nurtured in such environments aligns with the core characteristics of a growth mindset, which emphasizes effort, persistence, and learning from failure (Dweck, 2006). Moreover, a diverse and inclusive sports atmosphere, characterized by participation alongside peers of varied backgrounds, abilities, and experiences, promotes respect for differing perspectives and enhances social adaptability. Through continuous communication and collaboration, students develop greater interpersonal flexibility and resilience, key attributes associated with growth-oriented mindset (Fraguela-Vale et al., 2020). In addition, moderately challenging tasks paired with positive reinforcement from coaches and peers' recognition contribute to a heightened sense of competence and social acceptance (Bonaccorsi et al., 2020; Eather et al., 2023; Sakalidis et al., 2023). Furthermore, a team-oriented sports culture that emphasizes collective honor and shared goals fosters students' sense of belonging and social identity (Hubinette et al., 2014), reducing fear of failure and promoting the development of personal growth mindset.

It has been reported that individuals with growth mindset tend to adopt positive coping attitudes and strategies and are more likely to maintain an optimistic outlook when confronted with challenges (Dweck, 2006; Hecht et al., 2021; Zeng et al., 2016). Empirical studies consistently show that a growth mindset is linked to greater academic resilience, reduced psychological vulnerability, and better school adaptation and mental health (Duan et al., 2024; Zeng et al., 2016). For example, research on left-behind children demonstrates that cultivating a growth mindset enhances their psychological resilience and capacity to cope with adversity (Song and Xu, 2019). Similarly, studies among college students reveal that a growth mindset indirectly predicts wellbeing and anxiety levels by shaping stress perception and resilience (Jia et al., 2022), highlighting its critical role in fostering psychological resilience. Sports atmosphere, including intrinsic supportive factors such as sharing experiences with coaches and peers, joint training and competition, and adherence to rules and norms (Arufe-Giraldez et al., 2019; Dong and Mao, 2020; Liu et al., 2024), not only enhances students' sports skills but also transfers to other areas, helping them manage pressure, improve empathy, teamwork, and communication, and develop respect for rules and authority. Such experiences strengthen psychological resilience by fostering perseverance under stress. It has been reported that a positive sports atmosphere fosters higher levels of growth mindset and psychological resilience in college students, highlighting its role in cultivating adaptive psychological resources (Fleck et al., 2024; Gu et al., 2022; Liu et al., 2024). Nevertheless, the role of growth mindset in the influence of sports atmosphere on psychological resilience has not been elucidated. In the present study, our findings indicate that growth mindsets mediate the effects of sports atmosphere on psychological resilience. That is to say, sports atmosphere not only directly affects the college students' psychological resilience, but also indirectly affects psychological resilience of college students by a growth mindset. The above results broaden our understanding of the possible mechanism of the influence of sports atmosphere on the psychological toughness of college students and presents available strategy for the cultivation of college students' growth mindset and psychological resilience. However, due to the cross-sectional design of this study, the number of sample size, self-reported results and potential confounding factors could not be fully controlled, which may influence the mediating effect of growth mindset between sports atmosphere and psychological resilience. For instance, personality traits (Zhang et al., 2024), social support (Cohen and Wills, 1985), and prior mental health status (Jung et al., 2021) may affect both the independent and dependent variables, thereby compromising the validity of the mediating effects. Even though, the study yields important implications for understanding the psychological mechanisms underlying college students' development. The consistency of the findings with existing literature suggests that the observed relationships are meaningful and warrant further investigation in broader and more diverse populations.

This study provides valuable theoretical and practical insights for advancing higher education. It highlights the critical role of a positive sports atmosphere on campus, emphasizing the responsibility of administrators to invest in facilities, events, and policies that promote physical activity. Such efforts can enhance not only students' physical health but also their psychological resilience, supporting a more holistic approach to student wellbeing. Creating a campus culture that values and normalizes physical activity, offering diverse and inclusive sports programs, and integrating messages about personal growth and effort into physical education and wellness campaigns are suggested. By combining physical activity promotion with psychological development strategies, universities can effectively enhance students' resilience and adaptive capacities. Coaches can foster a growth mindset by emphasizing effort, perseverance, and learning from setbacks, while promoting a team culture of respect, inclusivity, and wellbeing. Likewise, counselors can integrate growth mindset principles into their practices, helping students reframe challenges as opportunities and build resilience through targeted workshops and sessions.

In addition, due to this study focused on college students in Shanghai, it is essential to recognize that the socio-cultural context may influence the relationship between sports atmosphere, psychological resilience, and growth mindset. Distinct cultural and policy systems contribute to psychological resilience by shaping stress responses and providing support through various resources and programs. For instance, collectivist cultures enhance group support, while education and public health initiatives bolster mental health and psychological resilience (Ungar and Theron, 2020). Different school environments and their emphasis on sports play a crucial role in the cultivation of psychological resilience. Sports colleges or universities that prioritize athletic development typically offer more resources and opportunities for student participation in sports. Conversely, vocational or academically oriented institutions may place less emphasis on sports participation, potentially undermining students' psychological resilience (Zhao, 2024). Future research should explore the impact of sports atmosphere on psychological resilience across diverse university environments and investigate how cultural attitudes toward sports and academic achievement shape this relationship. Cross-cultural and longitudinal studies are recommended to analyze these dynamics among various student populations and academic disciplines.

## 6 Conclusions and prospects

This study reveals that growth mindset serves as mediating factor between sports atmosphere and psychological resilience. Specifically, it suggests that the influence of sports atmosphere on psychological resilience may derive from its capacity to enhance college students' growth mindset. This finding not only deepens our understanding of how sports atmosphere affects psychological resilience among college students but also presents new empirical strategies for fostering their psychological resilience. Consequently, schools should aim to cultivate a positive sports atmosphere and encourage college students to actively engage in sports activities, thereby assisting them in maintaining resilience and adaptability in the face of future challenges.

However, this study also has certain limitations. The cross-sectional research design employed restricts our understanding of causal relationships. To more accurately ascertain the dynamic interplay between sports atmosphere, growth mindset, and psychological resilience, future studies should consider utilizing longitudinal tracking designs or experimental approaches to better capture the trajectories of these variables over time. Specifically, it is proposed to implement interventions aimed at creating a positive sports atmosphere, such as organizing diverse physical activities, providing encouraging sports environments, and promoting teamwork and positive interactions. Through long-term follow-up studies (e.g., 6 months or a year), we could observe how these interventions influence changes in students' psychological resilience. Future research could also focus on potential moderating and mediating variables, such as social support, emotion regulation strategies, and types and frequency of physical activities, to further clarify how a positive sports atmosphere, through the growth mindset, contributes to enhancing psychological resilience. Moreover, in longitudinal studies, we recommend collecting data at multiple time points to analyze the temporal evolution of the relationships between sports atmosphere, growth mindset, and psychological resilience. Techniques such as growth curve modeling or cross-lagged modeling could be employed to explore whether a positive sports atmosphere can consistently enhance a growth mindset and thereby promote psychological resilience, or whether there is a bidirectional dynamic relationship among the three. These insights could provide more precise theoretical foundations for designing effective interventions. Finally, a limitation of this study is the relatively small sample size, which may affect the generalizability of the findings. However, the robustness of the results, as analyzed through effect size and confidence intervals, suggests that the findings of this study hold practical significance. Furthermore, we recommend that future research should expand the sample size and employ more rigorous sampling methods, such as random sampling or stratified sampling, to further validate the research conclusions.

## Data Availability

The raw data supporting the conclusions of this article will be made available by the authors, without undue reservation.
